# Co-disposal of lignite fly ash and coal mine waste rock for neutralisation of AMD

**DOI:** 10.1007/s11356-021-13500-w

**Published:** 2021-04-29

**Authors:** Asif Qureshi, Christian Maurice, Björn Öhlander

**Affiliations:** 1 Department of Civil, Environmental and Natural Resources Engineering, Division of Geosciences and Environmental Engineering, Luleå University of Technology, SE-97187 Luleå, Sweden; 2 Department of Energy and Environment Engineering, Quaid-e-Awam University of Engineering, Science and Technology, Nawabshah, 67480 Pakistan

**Keywords:** Coal mine waste rock, Acid mine drainage, Fly ash, Co-disposal, Remediation, Prevention

## Abstract

Waste rocks (WRs) from a lignite-producing coalfield and fly ash (FA) produced from the same lignite have been investigated in this study with a primary objective to determine the potential for co-disposal of WRs and FA to reduce the environmental contamination. Mixing WRs with FA and covering WRs with FA have been investigated. Particle size effect caused ≤2 mm particles to produce low pH (~2) and metal-laden leachates, indicating higher sulphide minerals’ reactivity compared to larger particles (≤10 mm, pH ~ 4). Co-disposal of FA as mixture showed an instantaneous effect, resulting in higher pH (~3–6) and better leachate quality. However, acidity produced by secondary mineralisation caused stabilisation of pH at around 4.5–5. In contrast, the pH of the leachates from the cover method gradually increased from strongly acidic (pH ~ 2) to mildly acidic (pH ~ 4–5) and circumneutral (pH ~ 7) along with a decrease in EC and elemental leaching. Gradually increasing pH can be attributed to the cover effect, which reduces the oxygen diffusion, thus sulphide oxidation. FA cover achieved the pH necessary for secondary mineralisation during the leaching experiment. The co-disposal of FA as cover and/or mixture possesses the potential for neutralisation and/or slowing down AMD and improving leachate quality.

## Introduction

Despite the latest developments towards cleaner alternative fuels, coal still makes up to about 15.9% share in the total global primary energy supply (IEA 2019a). Worldwide coal production increased by 250 Mt (~3.3% compared to 2017) with a total of ~7.81 Bt in 2018 (IEA 2019b). However, coal mining raises particularly significant environmental concerns. Apart from the carbon dioxide effect, it produces waste rocks (WRs) that often contain sulphides, with principally high contents of iron sulphides such as pyrite (FeS_2_) and pyrrhotite (Fe_1 – x_S). High concentrations of trace elements such as As, Cu, Hg, Zn, Ni, Co, Mo, Se, and Cr are also often present in WRs.

The sulphide-rich WRs are considered environmentally damaging because the sulphide minerals are unstable when exposed to the surface atmospheric conditions. The sulphide minerals, in presence of water and oxygen, produce acidic leachates with increased concentrations of major and trace elements that eventually end up in natural water resources. Such a process is commonly referred to as acid mine drainage (AMD). For instance, results from Qureshi et al. (2016a) showed that the WRs from a lignite-producing coal field in Pakistan had strong AMD potential and could seriously affect the quality of the surrounding environment.

Therefore, there are many methods that have been developed to treat and/or mitigate AMD from mining wastes in the last few decades such as in-pit disposal, wet and dry covers, and blending and co-disposal (Li et al. 2011; Villain et al. 2013; Nason et al. 2013; Mäkitalo et al. 2015; Kalonji Kabambi et al. 2017; Pouliot et al. 2018; Jia et al. 2019). The prevention (minimisation) techniques for AMD aim to stop sulphide oxidation and migration of weathering products to the environment by minimising the reaction rates and elemental leaching (INAP 2014). The basic concept behind most of the prevention techniques is to reduce and/or stop oxygen diffusion and water infiltration through the waste deposits.

Although selection of prevention/mitigation methods can be site-specific, in-pit disposal, mixing and blending, co-disposal, and wet and dry covers are the most commonly used methods today. For instance, wet and dry covers have been studied by Boulanger-Martel et al. (2017), Höglund et al. (2004) and Lessard et al. (2018).

On the other hand, utilisation of coal for heat and power generates fly ash (FA) that contains ecologically harmful substances (including As, B, Cd, Cr, Cu, Pb, Se and Zn) (Martinello et al. 2014; Yao et al. 2015). Despite this, FA has been utilised in different ways such as in concrete/concrete products, blended cement, road base/sub-base and soil modification/stabilisation (Shanmugan et al. 2020; Gupta et al. 2020; Cavusoglu et al. 2021), thanks to its physical (self-hardening) properties. Moreover, in recent years, several studies have shown that FA and its composites are effective in the neutralisation of AMD (Gitari et al. 2006; Pérez-López et al. 2007, 2009; Prasad and Mortimer 2011; Bäckström and Sartz 2011; Jia et al. 2014; Jouini et al. 2020). Therefore, FA, due to its acid-neutralising and physical properties, can be used as a dry cover material over WR dumps to isolate them from the surface atmospheric conditions and prevent AMD generation, or it can be mixed with WRs to backfill underground coal mines and prevent or neutralise any AMD that might be generated by the WRs. Notwithstanding, it is important to study both materials on a laboratory scale before any full-scale application to determine the best practice to be adopted in the field to remediate the waste materials.

Therefore, WRs from one of the largest lignite-producing coal fields in Pakistan and FA from a coal-fired power station that incinerates coal from the same field were studied in this research. The physicochemical behaviour of the WRs and FA has been reported in our previous studies (Qureshi et al. 2016a, b). Furthermore, effects of co-disposing these WRs and FA have also been reported in Qureshi et al. (2019) whereby 70 g of very fine grained crushed WRs (≤1 mm) were mixed in three different FA:WR ratios (1:1, 1:3 and 1:5) to perform weathering cell experiments. Although the acidity was not completely neutralised during the experiments, the results indicated that 1:1 ratio performed very well in improving pH and leachate quality. This is interesting because the approximate ratio of WR to FA production tends to be 1:1 from the Lakhra coal field and the power station. Therefore, the objectives of this study were to (i) evaluate the impact of particle size on AMD generation and leachate quality, (ii) determine the effectiveness of co-disposal (mixing and cover) of lignite FA for AMD neutralisation and (iii) identify the potential for precipitation/co-precipitation and/or dissolution of the secondary minerals to take place in both cases using a thermodynamic geochemical model (PHREEQC).

## Materials and methods

### Materials

#### Waste rocks

The samples of WRs were collected from four deposits near three underground coal mines in one of the largest coal fields (Lakhra) in Pakistan. The estimated coal reserves in the field are approximately 1.3 Bt with qualities varying from lignite to sub-bituminous (LCDC 2020). The WR samples were selected based on previously determined acid-generating potential (Qureshi et al. 2016a). The details of the WR samples are shown in Table 1.

**Table 1:** Details of the WR samples

Sample designation	Sample location	Characteristics	Acid-generating potential (NNP)^a^
WR1	Mine 1	Two to 3 months old	−144 ± 112 CaCO_3_ tonne^−1^
WR2	Mine 1	Less than a week old	−70 ± 6 CaCO_3_ tonne^−1^
WR3	Mine 3^b^	Less than a week old	−492 ± 178 CaCO_3_ tonne^−1^

^a^After Qureshi et al. (2016a)

^b^WR from mine 2 was excluded here due to the similar behaviour as WR3

#### Fly ash

The FA was generated from a fluidised bed combustion (FBC) power station which burns lignite coal with some addition of limestone. The FA sample was collected from the filter bag house.

### Methods

#### Mineralogy and chemical composition

A detailed mineralogical and chemical characterisation of WRs and FAs has been described in our previous work (Qureshi et al. 2016a, b) and is summarised in Table 2 and Table 3, respectively.

**Table 2:** Mineralogy of the samples determined by XRD

Sample	Mineralogy
FA^a^	Iron (III) oxide (Fe_2_O_3_), quartz (SiO_2_), anhydrite (CaSO_4_) and magnesioferrite (Mg(Fe^3+^)_2_P_4_), quiklime* (CaO)
WR1^b^	Dominated by quartz (SiO_2_), arsenopyrite (FeAsS) and kaolinite (Al_2_Si_2_O_5_(OH)_4_), with variable amounts of pyrite (FeS_2_), calcite (CaCO_3_) ad gypsum (CaSO_4_ 2H_2_O)
WR2^b^	Dominated by pyrite* (FeS_2_), kaolinite (Al_2_Si2O_5_(OH)_4_), haematite (Fe_2_O3) and gypsum (CaSO_4_·2H_2_O), with variable amounts of quartz (SiO_2_)
WR3^b^	Dominated by pyrite (FeS_2_), quartz (SiO2) and kaolinite (Al2Si2O5(OH)_4_), with variable amounts of malladerite (Na2SiF_6_), spangolite (Cu6Al(SO_4_)(OH)_12_Cl·3(H_2_O)), franklinite (ZnFe_2_O_4_)

^a^After Qureshi et al. (2016b)

^b^After Qureshi et al. (2016a)

*Fresh analysis

**Table 3:** Major and trace element composition of the samples as determined by ICP-MS and ICP-AES analyses

Element	FA ^a,b^	WR1 ^a,c^	WR2 ^a,c^	WR3 ^a,c^	CC ^d^
Dry weight (%)	98.7 ± 0.1	90.53 ± 5.38	77.33 ± 0.12	83.93 ± 0.23	n.d
Si (% dw)	12.43 ± 0.42	8.33 ± 5.79	11.14 ± 2.04	9.54 ± 3.97	27.72
Al (% dw)	9.47 ± 0.37	6.84 ± 4.7	9.24 ± 1.72	6.26 ± 2.35	8.13
Ca (% dw)	3.92 ± 0.14	3.69 ± 5.64	0.44 ± 0.1	0.36 ± 0.06	3.63
Fe (% dw)	24.25 ± 1.55	3.9 ± 4.26	1.57 ± 0.42	10.07 ± 3.88	5
K (% dw)	0.42 ± 0.01	0.4 ± 0.25	0.41 ± 0.11	0.33 ± 0.16	2.59
Mg (% dw)	1.22 ± 0.6	0.51 ± 0.09	0.54 ± 0.01	0.28 ± 0.04	2.09
Mn (% dw)	0.05 ± 0	0.05 ± 0.07	0 ± 0	0.01 ± 0	0.095
Na (% dw)	1.19 ± 0.03	0.16 ± 0.09	0.21 ± 0.04	0.21 ± 0.02	2.83
P (% dw)	0.03 ± 0	0.02 ± 0.01	0.02 ± 0	0.02 ± 0.01	0.11
Ti (% dw)	1.11 ± 0.06	0.41 ± 0.31	0.57 ± 0.13	0.69 ± 0.34	0.44
LOI (% dw)	7.93 ± 0.1	n.d	n.d	n.d	n.d
S (% dw)	2.40 ± 0.20	10.7 ± 12	1.90 ± 0.15	11.30 ± 4.70	0.02
As (mg/kg dw)	7.49 ± 0.6	8.15 ± 8.13	0.3 ± 0.08	3.88 ± 0.73	1.8
Ba (mg/kg dw)	211 ± 8	98.53 ± 61.46	123 ± 22	83.33 ± 36.15	425
Be (mg/kg dw)	7.25 ± 0.4	2.3 ± 1.48	3.48 ± 0.42	1.84 ± 0.32	2.8
Cd (mg/kg dw)	0.97 ± 0.1	0.3 ± 0.04	0.22 ± 0.07	0.25 ± 0.12	0.2
Co (mg/kg dw)	88.6 ± 8	40.23 ± 26.07	15.43 ± 4.55	43.5 ± 21.88	25
Cr (mg/kg dw)	168 ± 7	67.63 ± 52.6	111 ± 20	101 ± 37	100
Cu (mg/kg dw)	119 ± 12	73.43 ± 53.26	101 ± 30	24.97 ± 2.04	55
Hg (mg/kg dw)	0.49 ± 0.1	0.22 ± 0.07	0.14 ± 0.02	0.1 ± 0.02	0.08
Ni (mg/kg dw)	155 ± 15	87.83 ± 38.94	50.27 ± 14.26	61.1 ± 30.57	75
Pb (mg/kg dw)	18.9 ± 2	14.54 ± 9.14	20.27 ± 5.02	8.88 ± 3.57	13
Sr (mg/kg dw)	1510 ± 61	302 ± 103	241 ± 10	126 ± 18	375
V (mg/kg dw)	339 ± 7	178 ± 139	256 ± 38	139 ± 47	135
Zn (mg/kg dw)	218 ± 24	70.6 ± 7.6	50.1 ± 3.9	49.67 ± 15.15	70
Zr (mg/kg dw)	277 ± 5	75.03 ± 56.56	119.7 ± 22	135 ± 50	165

*dw* dry weight, *n.d* not determined, *n/a* not applicable

^a^Mean ± standard deviation (*n* = 3)

^b^After Qureshi et al. (2016b)

^c^After Qureshi et al. (2016a)

^d^Continental crust after Krauskopf and Bird (1995)

Briefly, the mineralogy was determined using XRD and SEM; the chemical characterisation was achieved by analysing the samples using ICP-MS and ICP-AES following the methods described in the “Physicochemical characterisation of eluates” section below.

#### Column leaching experiment

Columns were prepared from plexiglas tubes, each of 25 cm in height, 5 cm diameter and sealed with a rubber qwick cap at bottom. A nylon filter of 1 μm was placed and small nipple was installed at the bottom to filter and extract leachate, respectively. Sample material was filled and naturally compacted to a maximum height of approximately 15 cm.

**Fig. 1:**
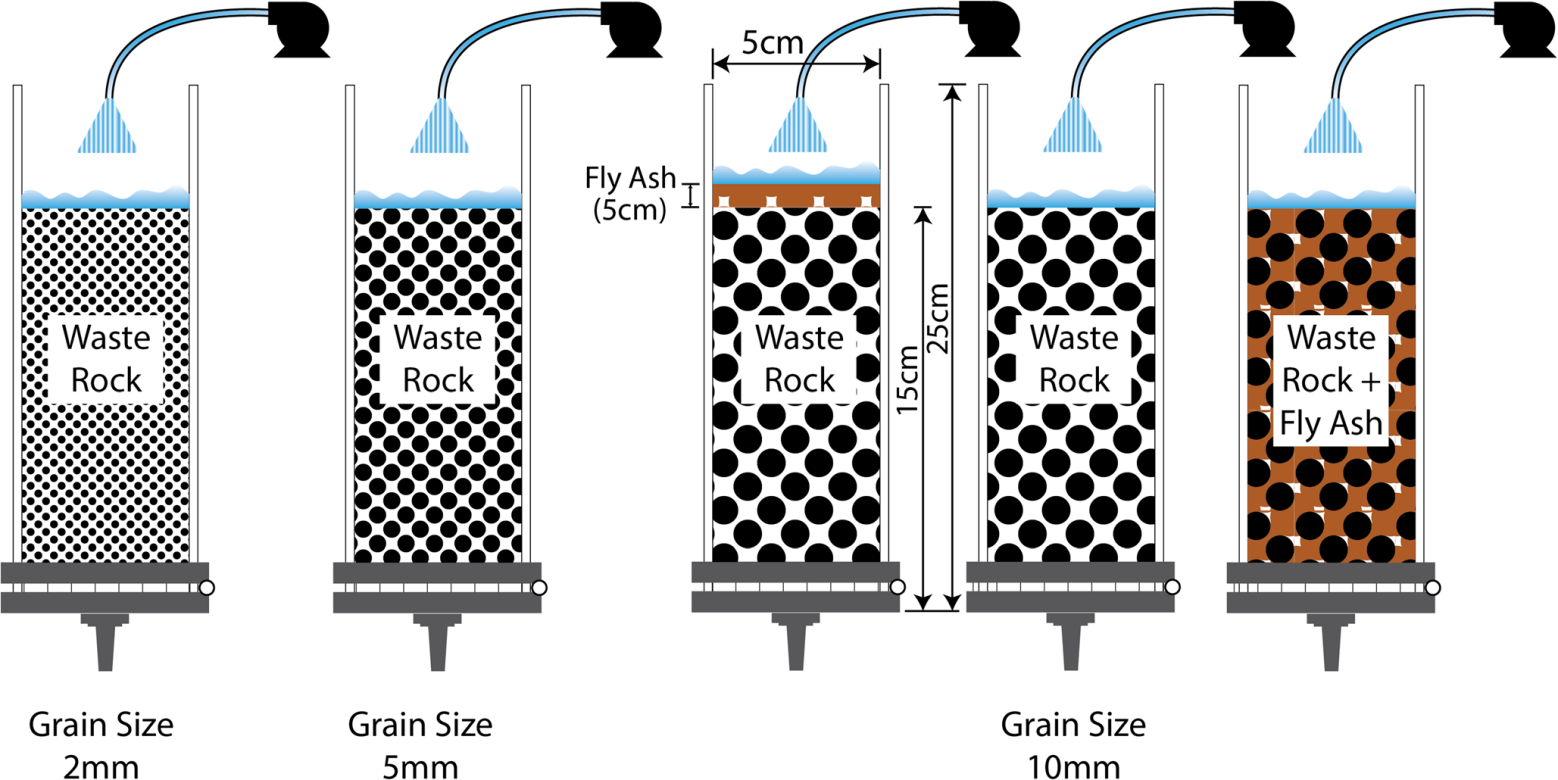
Illustration of column leaching experiment

Because this study also focuses on the effect of particle size on leaching, and due to the fact that our previous studies used very fine grain size of WRs (≤1 mm) for weathering cell experiments, the column leaching experiment (CLE) were performed on three different particle sizes of WRs (≤2 mm, ≤5 mm and ≤ 10 mm). The weight of material was 230 g of WR1, 265 g of WR2 and 278 g of WR3 in each column. FA cover and mixture scenarios were mimicked by making two separate columns with ≤10 mm particle size of WR sample, one with a small FA cover on top of the sample and one with mixing FA and WR. The proportion of FA (FA:WR) was 1:5 in the cover scenario and 1:3 in the mixture scenario. The ratios were inspired from our previous study (Qureshi et al. 2019). A total of five columns for each WR type were tested. An illustration of the experimental setup is shown in in Fig. 1.

Leaching was performed for 92 days (five rinses) with varying leaching times (1–4 days) and 10 drying days in between each rinse. Different liquid-to-solid (L/S) ratios (1, 5, 10 and 15 L kg^−1^) were used for each rinse, and, after the 4th rinse, the L/S ratio 1 was applied again to evaluate any effect of L/S ratio on differences in physicochemical composition of leachates. MilliQ™ water was used as leaching solution and a peristatic pump was used to control the flow rate and duration of each rinse.

Immediately after completion of each rinse, the eluates were filtered with a 0.45 μm nylon filter and the pH, redox potential (Eh) and electrical conductivity (EC) of each eluate were determined. The eluates were then placed in a laboratory freezer at a temperature below −18 °C before their chemical analyses. However, the effect of particle size on elemental leaching was only analysed for WR1. Since pH (Lottermoser 2007) and EC (Gray 1996a) can be good indicators of sulphide oxidation (AMD), they were used as indicators of AMD in WR2 and WR3.

#### Physicochemical characterisation of eluates

The proportions of 29 major elements (Si, Al, Ca, Fe, K, Mg, Mn, Na, P, Ti) and trace elements (As, Ba, Be, Cd, Co, Cr, Cu, Hg, Nb, Ni, Pb, S, Sc, Sr, V,W, Y, Zn, Zr) in the WRs and eluates were determined using inductively coupled plasma-atomic emission spectrometry (ICP-AES (Martin et al. 1991)) and inductively coupled plasma-mass spectrometry (ICP-MS (Long and Martin 1991)) at a SWEDAC-accredited laboratory (ALS Scandinavia, Luleå, Sweden).

The ICP-AES analyses were carried out using a Perkin Elmer Optima DV 5300 instrument following US EPA Method 200.7 (modified). The ICP-MS analyses were carried out using a Thermo-Scientific Element instrument following US EPA Method 200.8 (modified). Briefly, the samples were digested with HNO_3_ and analysed for As, Cd, Co, Cu, Hg, Ni, Pb, S and Zn. All other elements were analysed after fusion with lithium methaborate (LiBO_2_) and subsequent dissolution in HNO_3_.

Additionally, modified versions of procedures CSN EN ISO 10304-1 and CSN EN ISO 10304-2 for Cl, F and SO_4_^2−^ and CSN EN 1484 for DOC were carried out by the same laboratory.

#### Geochemical modelling

The results from the Physicochemical characterisation of eluates were used for geochemical modelling using the geochemical equilibrium model PHREEQC (Parkhurst and Appelo 2013) and MINTEQ.V4 database (Allison et al. 1991), to model the potential secondary mineral formation or dissolution.

A saturation index (SI) computed using PHREEQC indicated the thermodynamic tendency of a given mineral to precipitate or dissolve in the aqueous solutions. A negative SI indicated that the solution is not saturated with respect to a particular solid phase and that the solid phase would tend to dissolve if present, while a positive value indicated a tendency for the mineral to precipitate. A value close to zero suggested that the mineral was in equilibrium in the solution and may either precipitate or dissolve.

## Results and discussion

### Mineralogical and chemical composition

Since sulphide minerals are the most environmentally damaging minerals in mining waste (because of their unstable nature in surface atmospheric conditions and their potential for producing AMD), it was important to determine whether the WRs contained pyrite and other sulphide minerals. The XRD analyses carried out on the WRs (Table 2) revealed that they contained pyrite along with various other minerals (such as quartz, kaolinite, calcite and gypsum in WR1, kaolinite, haematite, gypsum and quartz in WR2 and quartz, kaolinite, malladerite, spangolite and franklinite in WR3). Therefore, the WRs were predicted to generate AMD, something which has been confirmed previously (Qureshi et al. 2019, 2016a). Minerals such as calcite and kaolinite can contribute towards the self-neutralising potential of WRs, depending on pH levels. However, buffering by kaolinite is much less efficient than that of carbonates (Ritchie 1989).

The mineral composition of FA (Table 2) was dominated by ferric oxide followed by quartz, anhydrite, magnesioferrite and lime. Conventionally, the Ca-bearing minerals are considered responsible for the acid-neutralising potential of FA (Gitari et al. 2006), though silicates, Fe– and Al–oxides can also contribute to acid-buffering processes in low pH conditions (Lottermoser 2007).

The WR samples contained high concentrations of Fe and S (Table 3), varying from 1.5 to 10 wt.% and 1 to 11 wt.%, respectively. The high sulphur content of the WRs is mainly due to the high sulphur coal in the field, as reported by Siddiqui (1999).

The concentrations of all the trace elements in the WRs were similar to their corresponding concentrations in the continental crust (CC; (Krauskopf and Bird 1995)), except for Ba (higher in CC), Cu (lower in CC) and Sr (higher in CC). In contrast, almost all trace elements were found enriched in the FA compared to CC, except for Ba (higher in CC) and Sr (lower in CC).

Despite the relatively low concentrations of trace metals, the high S content suggests that some of these elements such as Fe, Mn, As, Cd, Cu, Hg and Zn may occur in sulphide minerals in the WRs, and, under low pH conditions, become mobile. Nonetheless, Fe and other elements (such as Si, Al, Ca and Mg) may contribute to secondary mineral formation and help sorption to take place by binding elements (by co-precipitation and/or adsorption) together while forming these secondary minerals, depending on the pH levels (Lottermoser 2007).

### Impact of particle size on AMD and leachate quality

**Fig. 2:**
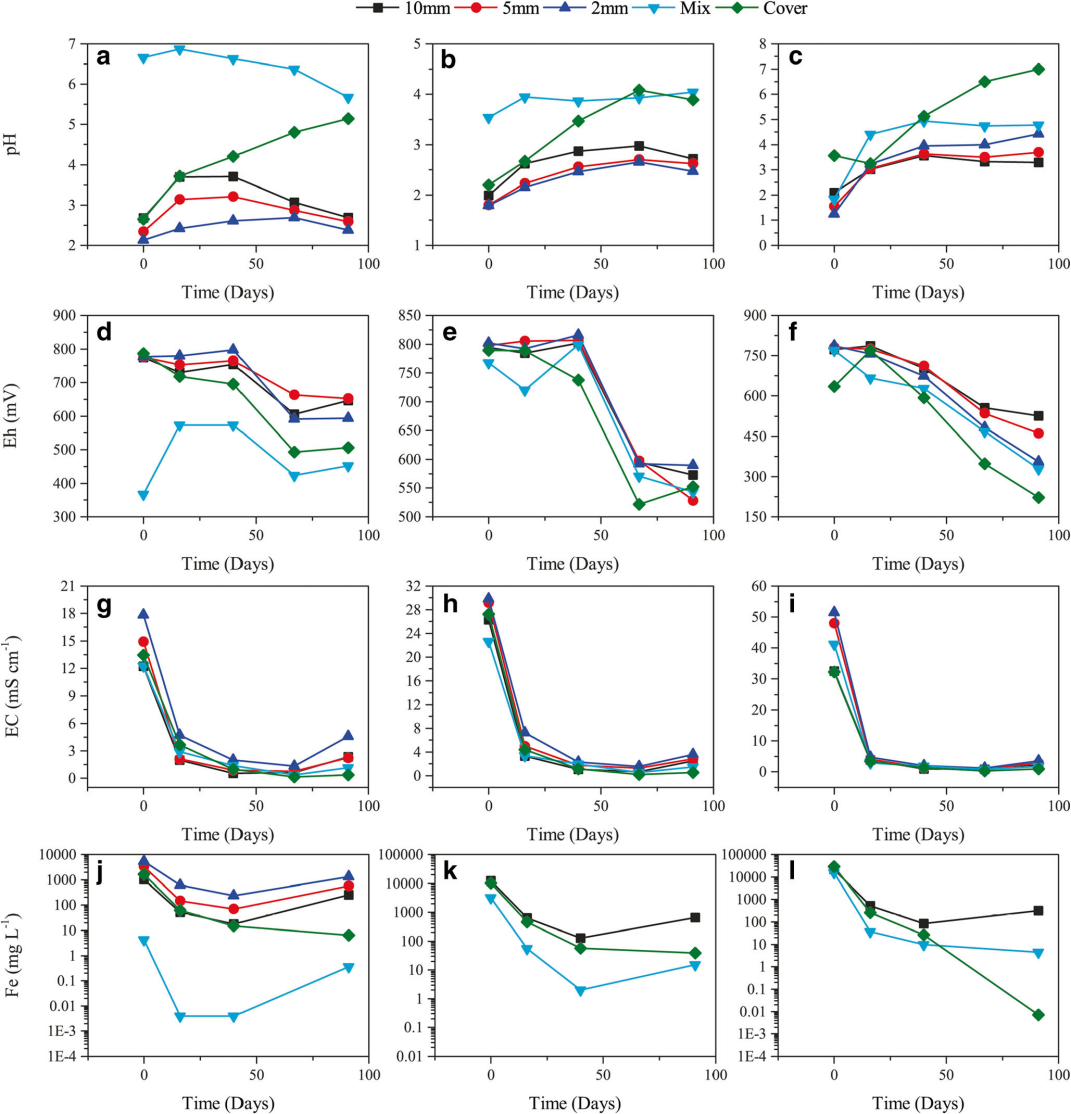

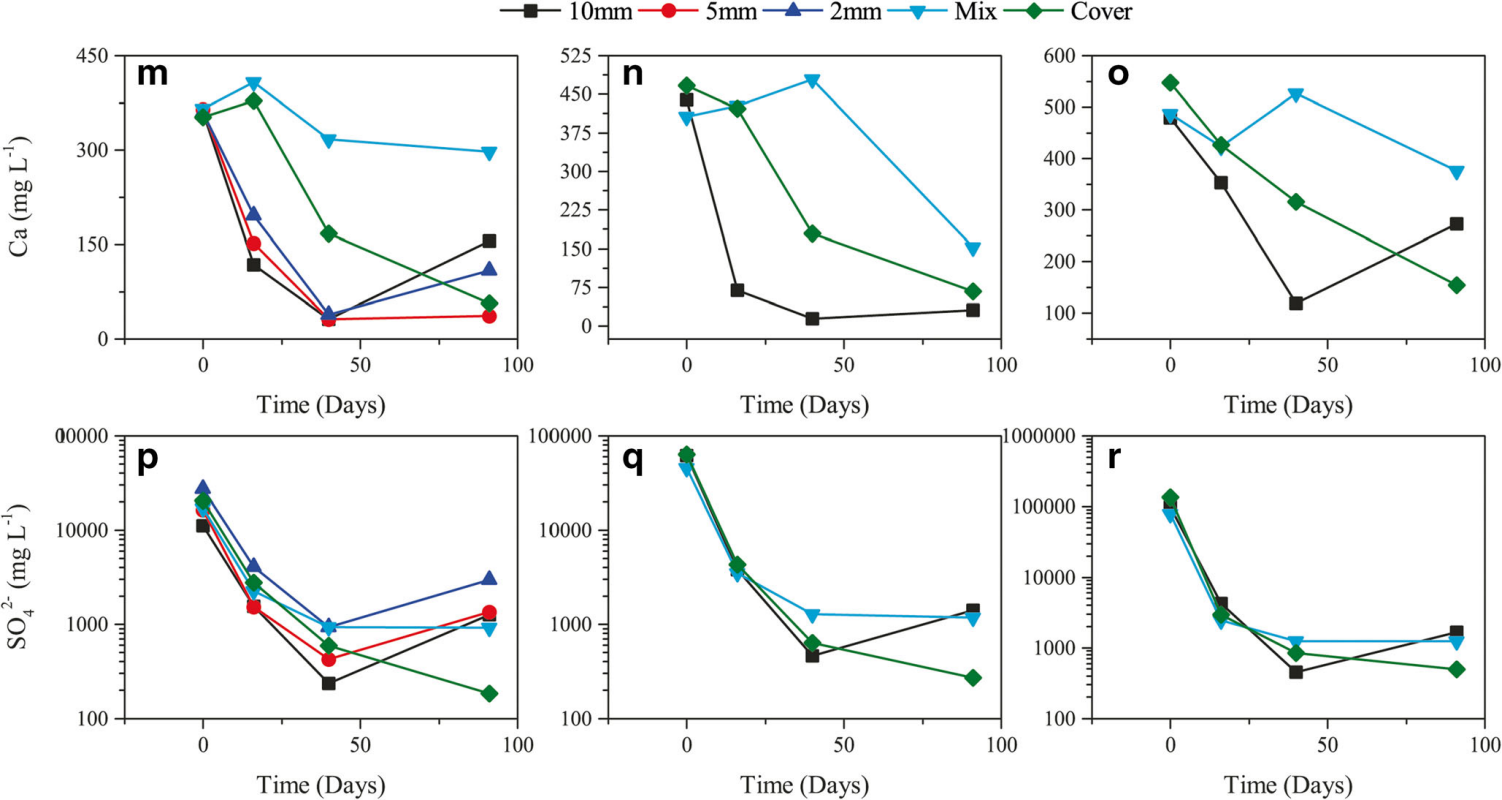
Physicochemical characteristics of the leachates from CLE. **a** pH from WR1. **b** pH from WR2. **c** pH from WR3. **d** Eh from WR1. **e** Eh from WR2. **f** Eh from WR3. **g** EC from WR1. **h** EC from WR2. **i** EC from WR3. **j** Fe from WR1. **k** Fe from WR2. **l** Fe from WR3. **m** Ca from WR1. **n** Ca from WR2. **o** Ca from WR3. **p** SO_4_^2−^ from WR1. **q** SO_4_^2−^ from WR2. **r** SO_4_^2−^ from WR3

The effect of the WRs’ particle size on pH, Eh and EC (thus, AMD) is visible in Fig. 2. Although the differences in pH were not large, there were some differences in EC from all three columns, being highest in the column with the smallest particles (≤2 mm).

Chemical composition of the leachates from the WR1 columns (Table 4) shows differences in leaching of elements (such as Fe, SO_4_^2−^, Al, Zn, Co, Cr, Cu, Mn and Ni) by varying the particle size of the samples. Such differences in concentrations indicate that the particle size is one of the driving forces behind the activity of AMD generating reactions and contamination of surrounding environment at mine sites.

**Table 4:** Minimum and maximum concentrations of the selected elements in eluates from WR1

Element	Sample	10 mm	5 mm	2 mm	Mixture	Cover
		Min–max	Min–max	Min–max	Min–max	Min–max
pH		2.68–3.71	2.34–3.21	2.13–2.61	5.67–6.87	2.65–5.14
Eh	mV	646.5–773.7	652.5–775.5	593.6–796.7	367.1–573.6	505.7–786.6
EC	mS/cm	0.535–12.21	0.891–14.94	1.975–17.84	1.159–12.28	0.378–13.44
Ca	mg/l	31.6–360	31.5–364	38.4–359	297–408	56.4–378
Fe	mg/l	17.6–1020	69.4–3320	239–5290	0.004–4.3	6.36–1640
K	mg/l	0.54–12	0.5–11.4	3–11.3	1.13–14.9	1.4–15.7
Mg	mg/l	25.2–1070	27.8–1440	24.9–1450	29.4–1840	7.06–1380
Na	mg/l	5.01–521	4.28–539	4.71–483	8.48–1390	7.48–1040
Cl	mg/l	5.87–464	7.78–552	8.99–506	1.89–993	2.55–876
F	mg/l	0.2–0.825	0.2–1.23	0.2–1.71	0.266–6.02	0.2–6.64
SO4^2−^	mg/l	235–11,200	425–16,500	929–27,200	916–17,100	183–20,600
DOC	mg/l	3.61–80.1	3.11–167	8.61–216	0.87–112	3.2–131
Al	mg/l	2.28–134	6.81–429	15.2–628	0.002–0.196	0.114–258
Zn	mg/l	2.13–108	2.06–198	1.59–203	0.661–10.6	2.11–67.1
As	μg/l	0.5–12	0.5–51.4	1.24–98.9	0.5–0.575	0.5–37.8
Ba	μg/l	4.61–43.9	3.16–38.5	4.97–36.6	25.4–46.7	10.6–36
Cd	μg/l	0.674–19.5	1.19–51.3	2.43–62.2	0.426–1.55	0.251–32.1
Co	μg/l	65.4–4490	132–8140	254–9540	31.7–1510	27.7–4580
Cr	μg/l	13.1–476	40.1–1820	107–2090	0.5–0.82	2.83–612
Cu	μg/l	19.7–514	44.1–1280	210–2160	1–3.86	6.4–1030
Mn	μg/l	261–6770	648–18,600	1140–24,900	341–14,600	184–10,600
Ni	μg/l	98.2–6170	212–11,600	469–14,400	42–2040	39.3–6750
Pb	μg/l	0.316–16	2.88–50.1	48.9–202	0.2–0.2	0.2–9.76

This is mainly because the smaller particle sizes possess larger surface area and expose more sulphide mineral surfaces to oxygen and water, thus accelerating AMD generation. However, a sudden spike in pH and dip in EC, Eh and elemental leaching from the WR1 columns from the first to the second rinse indicate an initial flush-out effect, something that has also been observed previously (Qureshi et al. 2016a).

Because EC is very sensitive to the concentration of dissolved salts and SO_4_^2−^ concentrations in water, it has been used as a preliminary indicator of AMD in some studies (Gray 1996b; Lyew and Sheppard 2001; Galhardi and Bonotto 2016). Therefore, elevated EC (Fig. 2 g, h and i) and lower pH (Fig. 2 a and b; except WR3) compared to columns with larger particles can indicate higher AMD generation in columns with smaller particle sizes. Moreover, elevating pH trends in the column with smallest particles of WR3 can also indicate that smaller particle sizes also expose more acid-buffering minerals (which might have precipitated due to natural weathering or secondary mineralisation during storage of the WR samples) which consume acidity, when the pH of the system is acidic (Lottermoser 2007; Sánchez España 2007). Higher pH along with higher EC from the column with smallest particles of WR3 also indicate that secondary mineral formation is occurring slower in this column than other columns, which was not observed before during experiments performed on particles ≤1 mm for the same WR, and even with a smaller (FA:WR, 1:5) addition of FA (Qureshi et al. 2019).

Despite the chemical composition of the leachates from the WR2 and WR3 columns were not determined, their comparative pH and EC with WR1 can be indicative of the sulphide reactivity and elemental leaching. Furthermore, due to dependence of element mobility on pH, lower pH conditions and higher EC also suggest that the elemental concentrations would also be high in the leachates from WR2 and WR3.

### Impact of co-disposal of FA and WRs on AMD and leachate quality

#### Co-disposal by FA and WR mixture

FA addition has shown to have considerable influence on pH and other physicochemical characteristics of the leachates regardless of the FA addition method (Fig. 2 and Tables 4 and 5), but the efficiency differed between the methods and the WR samples. For instance, in the mixture scenario in WR1, pH of the leachate is always circumneutral, and, due to the fact that pH plays a crucial role in elemental leaching (Lottermoser 2007; Izquierdo and Querol 2012), elements associated with sulphide minerals (such as Fe, Al, Zn, As, Cd, Co, Cr and Cu) were always relatively low except on the first rinse where the flush-out effect took place.

**Table 5:** Minimum and maximum concentrations of the selected elements in eluates from WR2 and WR3

Element	Sample	WR2 (10 mm)	WR2 (mix)	WR2 (cover)	WR3 (10 mm)	WR3 (mix)	WR3 (cover)
		Min**–**max	Min**–**max	Min**–**max	Min**–**max	Min**–**max	Min**–**max
pH		1.99–2.87	3.54–4.04	2.2–4.08	2.09–3.56	3.56–4.93	1.84–7
Eh	mV	572–801	543–798	552–788	526–786	328–770	222–769
EC	mS/cm	1.08–26.3	1.483–22.62	0.533–27.23	0.995–32.51	1.73–41.2	0.92–32.29
Ca	mg/l	15–439	153–479	67.6–467	119–479	376–526	154–547
Fe	mg/l	128–12,500	2.04–3160	38.6–10,300	83.1–21,800	4.54–15,800	0.01–29,000
K	mg/l	3–10	0.635–10	0.5–14	0.5–43.4	0.5–10	0.5–55.9
Mg	mg/l	8.67–2040	6.74–3110	3.55–2400	40.6–1460	25.2–2920	25.2–2920
Na	mg/l	2.07–667	10.2–1870	5.83–1410	2.93–616	7.27–2110	7.27–2110
Cl	mg/l	4.34–706	1.69–946	2.69–886	2.41–324	1–624	1–624
F	mg/l	0.2–3.55	0.2–90.4	0.2–17.6	0.2–8.19	0.2–78.3	0.2–78.3
SO42-	mg/l	464–61,600	1180–45,700	269–63,600	455–98,100	1240–79,200	1240–79,200
DOC	mg/l	4.75–272	3.1–184	2.22–258	2.44–233	0.52–183	0.52–183
Al	mg/l	8.52–2200	25–2340	1.96–2180	5.11–3220	1.23–4250	1.23–4250
Zn	mg/l	1.07–287	1.78–218	0.395–191	0.574–266	0.74–214	0.74–214
As	μg/l	0.589–447	0.5–15	0.5–477	0.5–297	0.5–274	0.5–460
Ba	μg/l	2.74–26.9	21.2–59.7	10.5–19.4	9.09–58.4	28.9–43.2	4.49–45.5
Cd	μg/l	1.56–151	2.31–114	0.847–135	0.468–109	0.61–99.6	0.05–140
Co	μg/l	130–24,500	96.8–21,100	28.2–22,300	39.5–10,600	20–12,300	0.86–11,400
Cr	μg/l	57.6–5510	6.34–1460	10.5–4570	15.3–6460	0.5–3060	0.5–8390
Cu	μg/l	173–11,100	49.7–2500	28–9610	19.3–2720	3.95–867	1–2330
Mn	μg/l	450–49,500	243–47,600	147–46,200	501–55,500	374–72,500	294–68,400
Mo	μg/l	0.5–30.7	0.5–10	0.5–45.3	0.5–50.9	0.5–108	0.5–77.8
Ni	μg/l	242–36,000	194–30,200	72.1–31,100	50.1–12,000	32.3–15,700	1.61–14,200
Pb	μg/l	16.7–1770	0.2–4	2.56–1370	0.371–899	0.2–20	0.2–1080

**Fig. 3:**
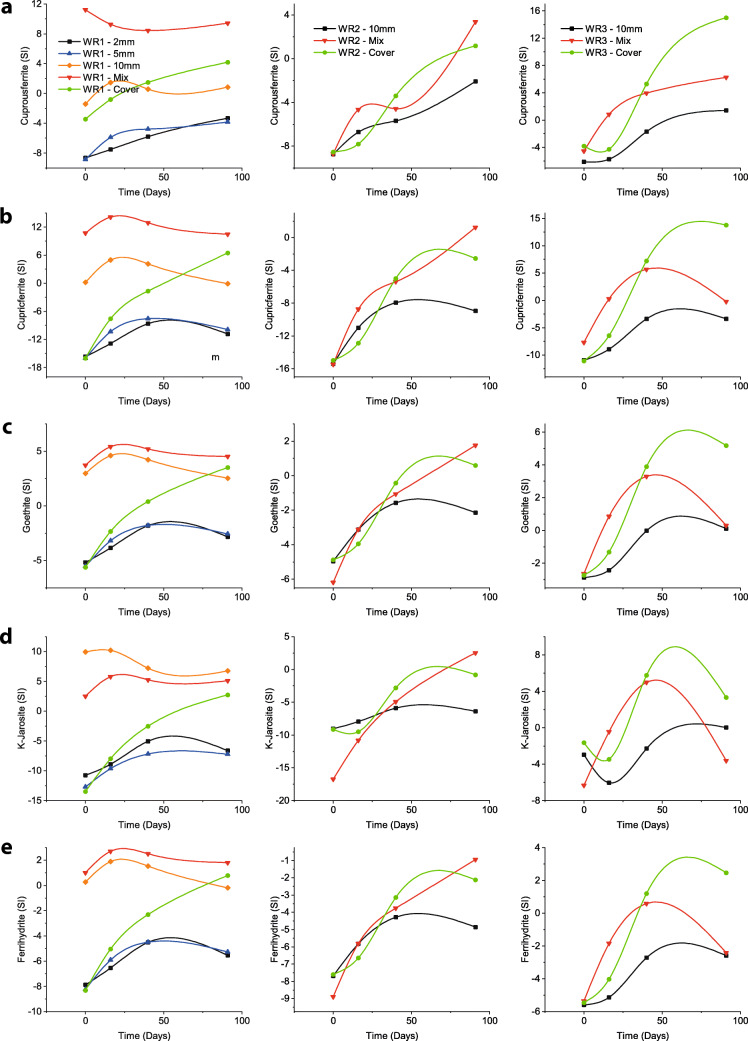

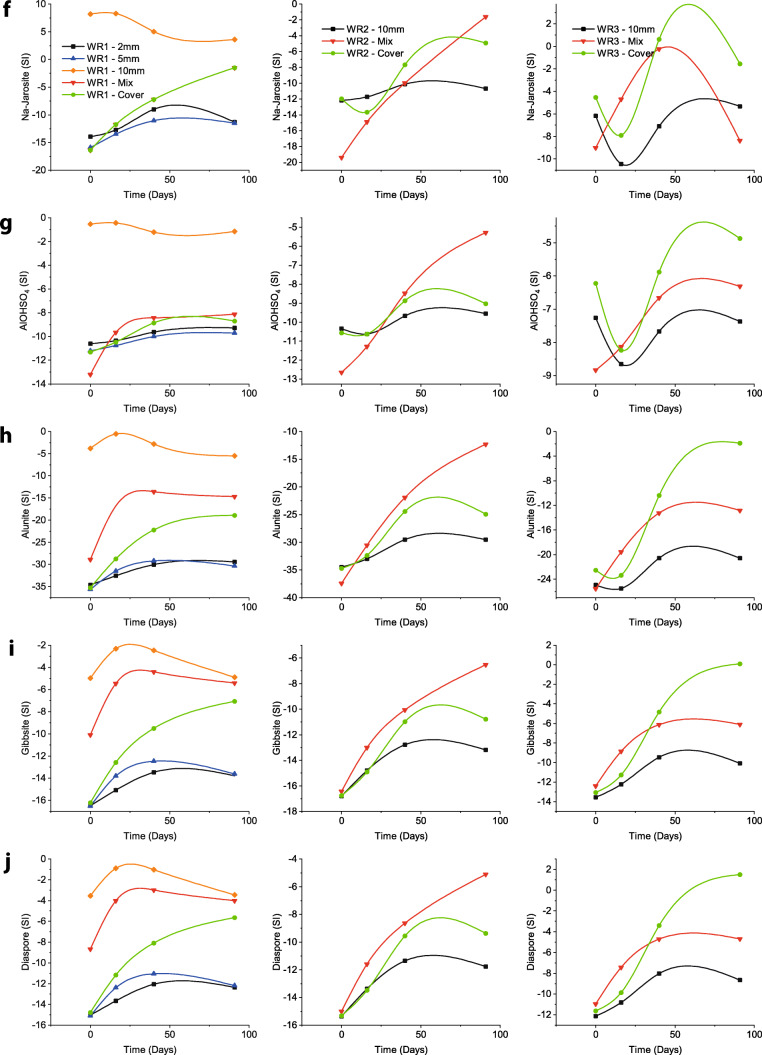
Saturation Index (SI) for selected minerals as computed by PHREEQC (left, WR1; centre, WR2; and right, WR3). **a** Cuprousferrite. **b** Cupricferrite. **c** Goethite. **d** K-Jarosite. **e** Ferrihydrite. **f** Na-Jarosite. **g** AlOHSO_4_. **h** Alunite. **i** Gibbsite. **j** Diaspore

However, a decreasing pattern of pH over the duration of the experiment has been observed in the same (mixture) column in WR1. This indicates that the secondary mineralisation (such as precipitation of goethite and ferrihydrite; Fig. 3) contributes towards the acidity of the solution (Lottermoser 2007). This could also indicate that the neutralisation provided by FA is not enough for complete neutralisation of the acidity produced by the reactivity of sulphide minerals and by secondary mineralisation. This may sometimes happen due to depletion of acid-neutralising minerals supplied by FA or when they become non-reactive due to secondary mineral coating (encapsulation) on their surfaces (Lottermoser 2007). The latter is probably not the case here, as indicated by presence of Ca in solution (Fig. 2m).

In general, the leachates from WR2 were constantly acidic regardless of the method of FA addition throughout the test duration, but small differences in pH (higher), EC (lower) and pronounced leaching of elements (such as Fe, Ca, Mg, Na, Cl and F) with FA addition compared to the columns without FA are visible in Fig. 2 and Table 5.

Leachates from the column with FA mixture in WR2 started with low pH (~3.5) and stayed acidic throughout the test duration with pH ~ 4 on the conclusion of the test. Moreover, this was twice as high as compared to columns without FA addition, indicating that the buffering reactions are taking place but their reactivity is slower compared to acid-producing reactions (Alakangas et al. 2013). Despite such low pH conditions from this column, FA addition stabilised pH between 3.5 and 4 which is enough for the precipitation of Fe^3+^ secondary minerals (or lowering their dissolution; Fig. 3) and removal of Fe from the solution (Wei et al. 2005; Balintova and Petrilakova 2011) (see Fig. 2k). However, acidity produced by secondary mineralisation (Lottermoser 2007) can also lower the pH of the solution and that might also be a reason behind constantly acidic leachates from this column.

WR3 is the most acidic in the collection of WR samples in this study (Table 1 and Qureshi et al. (2016a)). Leachates from the mixture column in WR3 start with acidic pH (~2) but get elevated to around pH ~ 4.4 on the second rinse and constantly remain in the range pH ~ 4.7–4.9 (Fig. 2 and Table 5). Sudden elevation in pH can be attributed to FA which is available within the pores between WR particles and buffers acidity as soon as it is produced. Despite that, the low pH conditions are probably because the pH is constantly within the range suitable for Fe^3+^ precipitation (Wei et al. 2005; Balintova and Petrilakova 2011) which produces more acidity (Lottermoser 2007) than the buffering minerals can consume, resulting in pH stabilisation after the 2nd rinse. Moreover, dissolution of Fe^3+^ and Al minerals as indicated by PHREEQC (Fig. 3) can be associated to their capability of acting as buffers when pH conditions tend to be acidic (Lottermoser 2007; Sánchez España 2007).

#### Co-disposal by FA cover

The cover scenario in WR1 has been quite different than mixture in every aspect (Fig. 2 and Table 4). It starts with low pH conditions (~2.6) and keeps improving until 5th rinse (~5.1). Such changes in pH indicate that the available oxygen within the column depletes with time and oxygen diffusion has been restricted by the FA cover on top, which usually happens when applying dry covers (Höglund et al. 2004; Mäkitalo et al. 2016).

Alternatively, the preferential flow, which causes high pH water (produced by FA cover on top) to pass through certain pathways throughout the column without interacting with the whole material, could also be a reason behind higher pH and lower elemental concentration in the leachates. However, this does not seem to be a case here, as indicated by constantly decreasing SO_4_^2−^, Ca, Fe, EC and Eh, while increasing pH (Fig. 2).

The cover column from WR2 starts with low pH (~2) and improves rapidly with maximum pH ~4 and 3.8 on the concluding day of the test. The lowering of pH from ~4 on the 67th day of the test to ~3.8 on the 91st day is probably due to secondary mineralisation within the column which produces acidity (Lottermoser 2007) since the Fe^3+^ minerals start to precipitate at pH ≥3.5 (Wei et al. 2005; Balintova and Petrilakova 2011). This has also been observed in Fig. 2k by constantly decreasing Fe concentrations in solution. EC of the leachates was little higher compared to the mixture column in first two rinses but stays relatively low afterwards. Flush-out effects may be reason behind such high EC, but it can also be due secondary mineralisation and elevating pH conditions that restrict elemental mobility.

Utilisation of FA as cover has been effective to neutralise strong acidity and/or prevent further acidity within the test duration and restrict element mobility to considerable extent from WR3. In the beginning, it produces strongly acidic leachates but pH rapidly and constantly elevated being highest at the conclusion of the test (pH ~ 7; Fig. 2c). Due to constant improvement (elevation) of pH, leaching of the elements has also been decreasing.

FA cover performs better than mixture, probably because (with time), similar to its counterpart in WR1, it restricts diffusion of fresh oxygen into the column, which slows down the weathering reactions on overall and results in constant increments in the pH and reduces elemental mobility (Fig. 2). It can also be because the FA provides acid-buffering minerals (and high pH waters) slowly and constantly which allows buffering reactions to take place consistently rather than rapidly. The slow release of buffering minerals and their transport down into the column can allow more time for the buffering reactions to take place effectively (Fig. 3). Furthermore, despite the secondary mineralisation (especially precipitation of Fe^3+^-hydroxides and -oxyhydroxides; Fig. 3) and decreasing Ca^2+^ (Fig. 2), the incremental pH can be indicative of the additional buffering supplied by the secondary Fe (such as jarosites tend to dissolve at the end; Fig. 3) and Al minerals (constant dissolution of AlOHSO_4_, alunite, gibbsite and diaspore; Fig. 3).

### Prospects of FA and WR co-disposal

Despite the FA used here possessed significantly less acid-neutralising potential compared to a biomass FA (20 kg CaCO_3_ tonne^−1^ compared to 275 kg CaCO_3_ tonne^−1^, respectively) in our previous study (Qureshi et al. 2016b), its addition has been considerably effective for neutralisation and/or reduction in AMD from the WRs during the CLE.

The WRs also behaved differently in our previous study (Qureshi et al. 2016a); for instance, WR1 produced circumneutral pH leachates during a weathering cell experiment for 192 days, probably because the chemical composition of the WR1 (*n* = 3) varies significantly as indicated by its Ca (3.69 ± 5.64), Fe (3.9 ± 4.26) and S (10.7 ± 12) content (Table 2; (Qureshi et al. 2016a)). However, the leachates from WR2 and WR3 were mildly (pH ~ 2.7–4.9) and strongly (pH ~ 0.9–2.3) acidic, respectively, in our previous study (Qureshi et al. 2016a).

It has been observed from the results that co-disposal by mixture works almost instantaneously (indicated by slightly higher pH and lower EC; Fig. 2), but it seems that the secondary mineralisation (especially secondary Fe and Al minerals) dominate the system over time and contributes towards acidity, which causes pH stabilisation to ~4.5–5. Such secondary mineralisation can remove Fe^2+^/Fe^3+^ from the solution, bind toxic elements (such as As, Pb, Cu, Zn, Cd, Co, Ni and Mn) and act as buffers when pH conditions tend to be more acidic (Lottermoser 2007; Sánchez España 2007). Therefore, co-disposal by mixture can be considered a rapid and effective method for disposing FA and WRs where materials are not strongly acid-generating and need additional buffering capacity to improve their environmental performance.

Utilising FA as cover material for WR dumps, on the other hand, behaves differently from the mixture. Because FA is available on the top (covering surface), the acidity generated from the WRs deep down in the columns is not neutralised by FA in the beginning (indicated by low pH and high EC, Fig. 2), but by Fe and Al minerals instead due to acidic pH conditions (Fig. 3; (Lottermoser 2007; Sánchez España 2007)). Furthermore, the cover effect (reducing oxygen migration) seems to take place which reduces sulphide minerals’ reaction rate and supplies acid-neutralising capacity gradually, causing gradual rise in pH. Such gradual changes in pH conditions also help the secondary minerals to precipitate and/or reduce their dissolution. The acidity produced by secondary mineralisation is not affecting the overall pH of the system because the reactivity of the acid-producing reactions (oxidation of sulphide minerals) has been reduced due to the depletion of available oxygen within the system and slower supply of fresh oxygen because of the cover effect.

Although FA has been effective for neutralisation and/or slowing down AMD generation, its addition pronounces concentrations of elements in leachates that are mostly enriched in FA (such as Ca, Na, Cl, F, Mo, Ba and SO_4_^2−^). Therefore, FA’s utilisation for the remediation, mitigation, neutralisation or prevention of AMD warrants special attention because mobility of many elements is pH-dependant and if the pH conditions tend to be acidic, FA might contribute in additional contamination. Notwithstanding, the results of this study support the co-disposal of FA with acid-generating WRs to improve AMD situation at the Lakhra coal field.

## Conclusions

The effect of particle size has been clearly observed in WR1 where smaller particle sizes produced leachates with elevated concentrations of the sulphide related elements (such as Al, Co, Cr, Cu, Fe, Mn, Ni, SO_4_^2−^ and Zn), indicating that sulphide oxidation has been pronounced in smaller particles. Similarly, low pH leachates with high EC from the other two WRs also indicate that the columns with smaller particle sizes of WRs 2 and 3 possess higher AMD-generating potential. However, slightly higher pH leachates from the smaller particle size WR3 columns were probably due to presence of some Fe and Al minerals that neutralise the pH, resulting in higher EC.

Co-disposal of FA as mixture readily provided acid-buffering minerals, resulting in better start-up pH conditions and leachate quality, compared to WRs alone. However, acidity produced by secondary mineralisation contributed towards the acidification of the system, causing stabilisation of pH at around 4.5–5. In contrast, the pH of the leachates from the FA cover scenario gradually increased from strongly acidic to mildly acidic (WR1 and WR2) and circumneutral (WR3) along with decrease in EC and elemental leaching. Gradually increasing pH was caused by the cover effect, which reduced oxygen diffusion, thus reducing sulphide oxidation, and causing pH to elevate. Because pH ~4–5 is sufficient for secondary Fe and Al mineral precipitation (which also removes toxic elements), the FA cover performs well to achieve this pH until the conclusion of the CLE. However, due to slower supply of acid neutralisation into the system, acidity produced in the beginning could not be neutralised.

The co-disposal of FA as cover and/or mixture possesses potential for partial neutralisation and/or slowing down AMD production and improving leachate quality. However, both systems need to be scaled up and investigated for AMD neutralisation, leachate quality and geotechnical stability by long-term experiments and kinetic modelling.

## Data Availability

All data generated or analysed during this study are included in this manuscript.
